# Supplementary material for: Genome Wide Identification and Annotation of NGATHA Transcription Factor Family in Crop Plants
Source: Int J Mol Sci. 2022 Jun 25;23(13):7063. doi: 10.3390/ijms23137063 (PMC9266525; doi:10.3390/ijms23137063)
Supplement: Supplementary file 1 [file ijms-23-07063-s001.zip › Supplementary figures _IJMS.pdf]

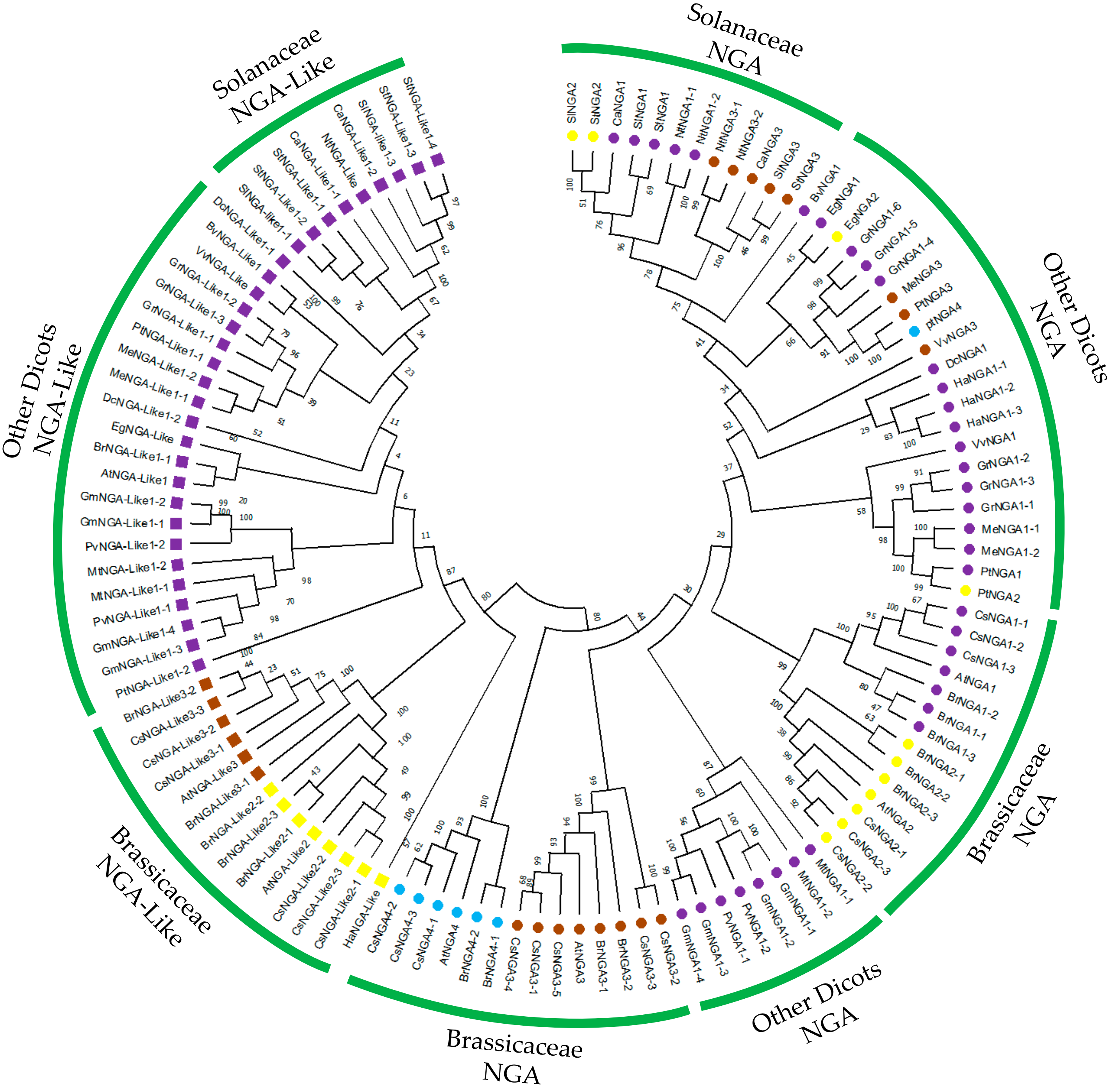


**Figure S1**. Phylogenetic analysis of NGATHA family in dicot plants. The phylogenetic tree was constructed using neighbor-joining (NJ) method with 1000 bootstrap replications in MEGA 11. The NGATHA proteins are divided into distinct families into Brassicaceae, Solanaceae and other dicot species. The symbols are represented to each of the NGATHA proteins as follows. ● NGA1, ● NGA2, ● NGA3, ● NGA4, ● NGA5, ■ NGA-Like1, ■ NGA-Like2, ■ NGA-Like3

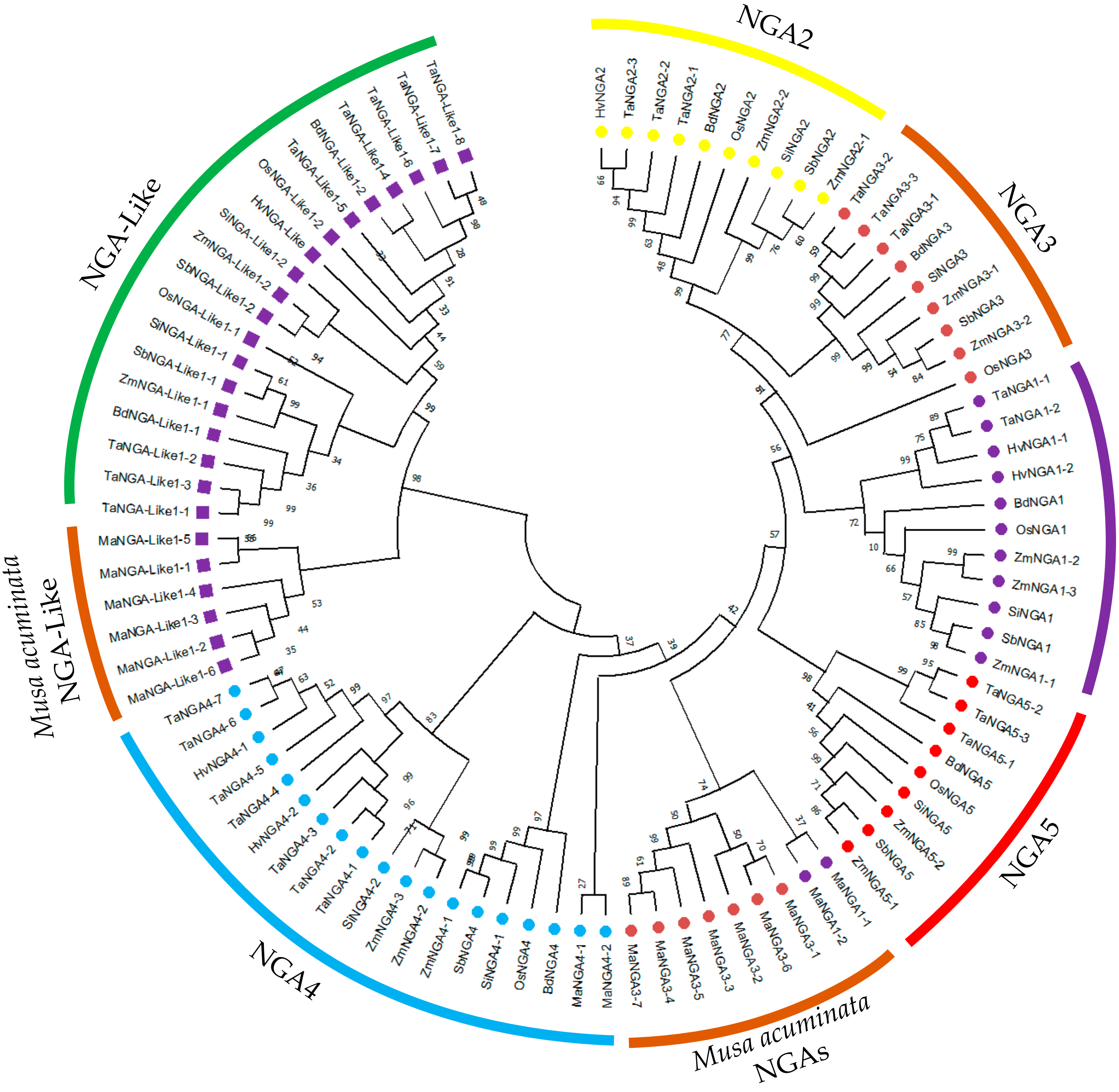


**Figure S2**. Phylogenetic analysis of NGATHA family in monocot plants. The phylogenetic tree was constructed using neighbor-joining (NJ) method with 1000 bootstrap replications in MEGA 11.The NGATHA proteins are divided into distinct subfamilies with five NGA proteins and NGA-Like proteins. The symbols are represented to each of the NGATHA proteins as follows. ● NGA1, ● NGA2, ● NGA3, ● NGA4, ● NGA5, ■ NGA-Like1, ■ NGA-Like2, ■ NGA-Like3

3a

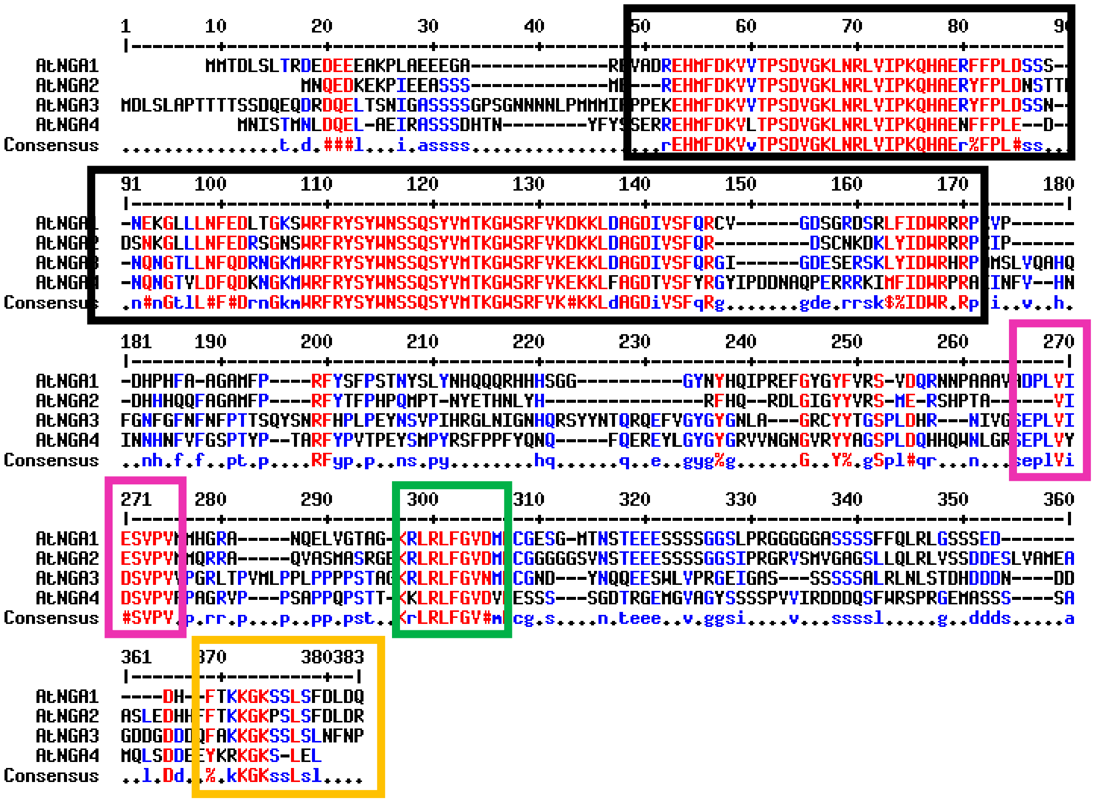
AtNGA1
AtNGA2
AtNGA3
AtNGA4
Consensus

3b

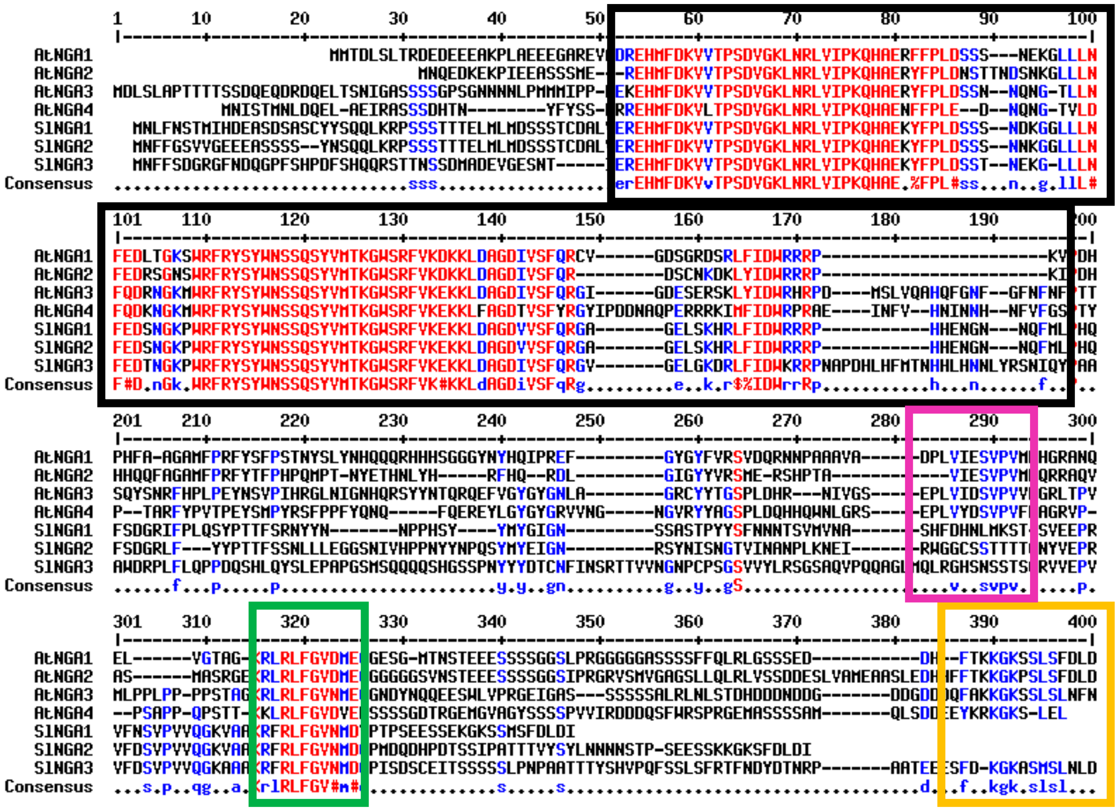
AtNGA1
AtNGA2
AtNGA3
AtNGA4
SlNGA1
SlNGA2
SlNGA3
Consensus

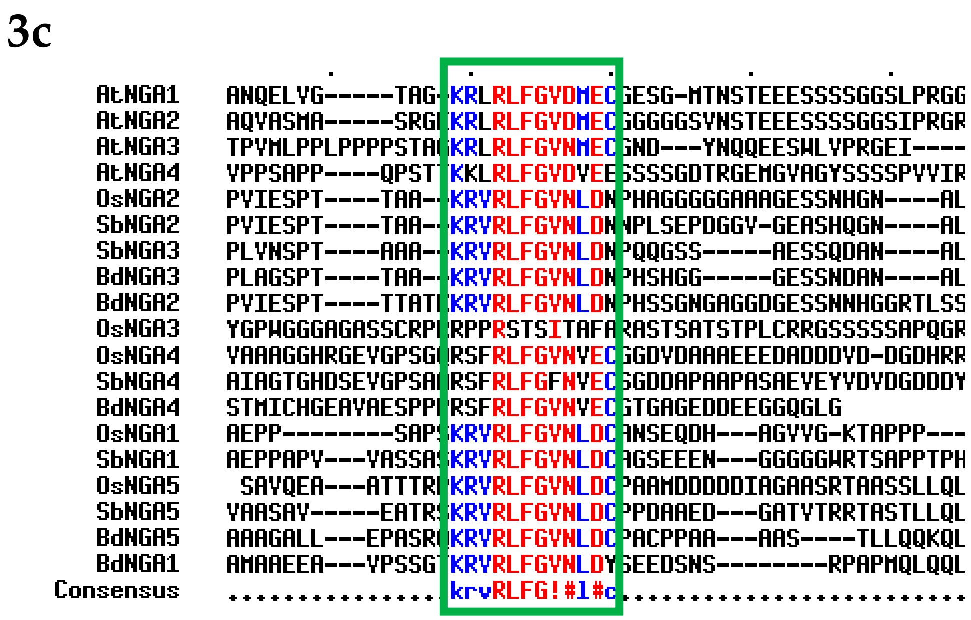

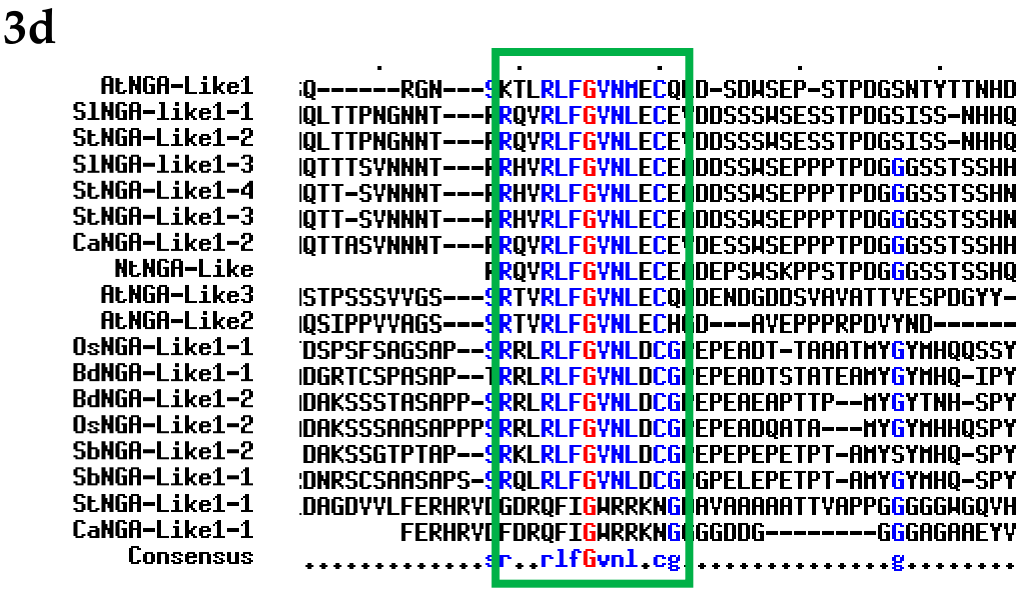

**Figure S3**. Multialignment of NGATHA proteins of (a) Arabidopsis; (b) Arabidopsis and tomato; (c) NGATHA proteins of Arabidopsis, rice, sorghum and Brachypodium; (d) NGATHA-Like proteins of Arabidopsis, rice, sorghum and Brachypodium, tomato, potato, capsicum and nicotiana.
The B3 domain is represented in black boxes. The repressor motif RLFGV is marked in green boxes. The repressor motif RLFGV is marked in green boxes. NGA-I and NGA-II motifs are indicated in pink and yellow boxes respectively.

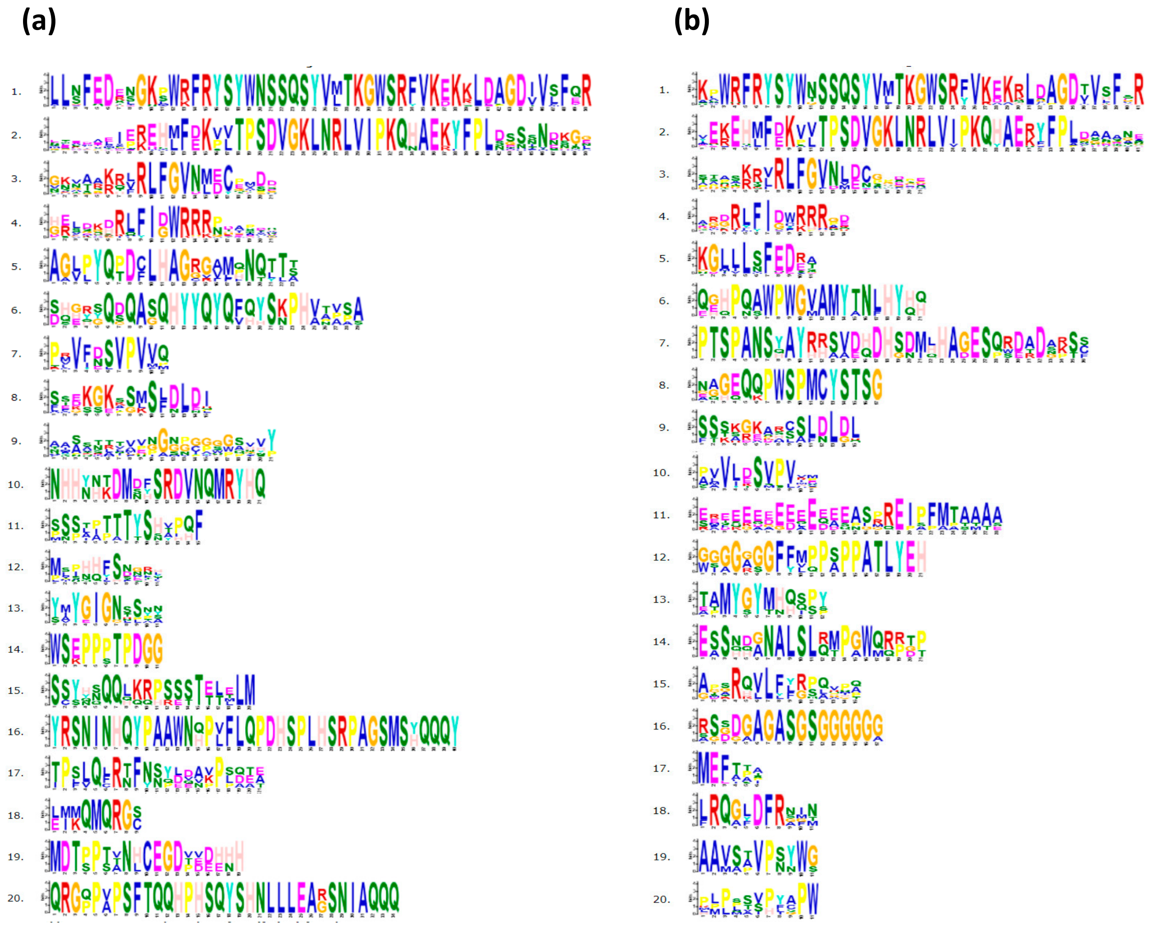


**Figure S4**. Protein motifs in the NGATHA proteins of (a) dicots, and (b) monocots

# TMHMM result

---

```
# WEBSEQUENCE Length: 4478
# WEBSEQUENCE Number of predicted TMHs:  0
# WEBSEQUENCE Exp number of AAs in TMHs: 27.9725499999998
# WEBSEQUENCE Exp number, first 60 AAs:  0.06739
# WEBSEQUENCE Total prob of N-in:        0.01256
WEBSEQUENCE     TMHMM2.0        outside      1   4478
```

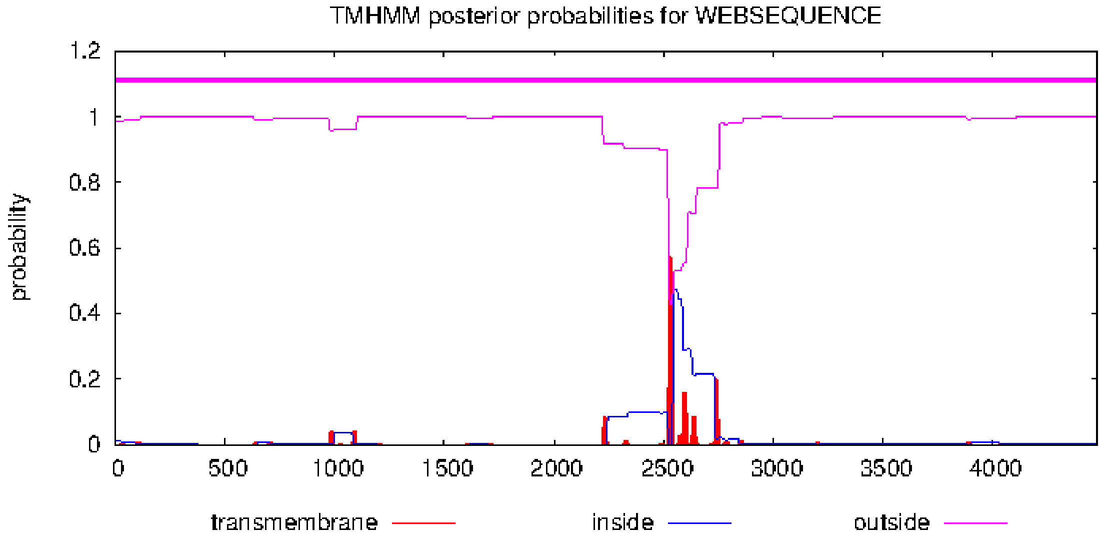


**Figure S5**. Analysis of  transmembrane domain in the NGATHA proteins. The TMHMM analysis predicts that there are no domains embedded within the membrane.

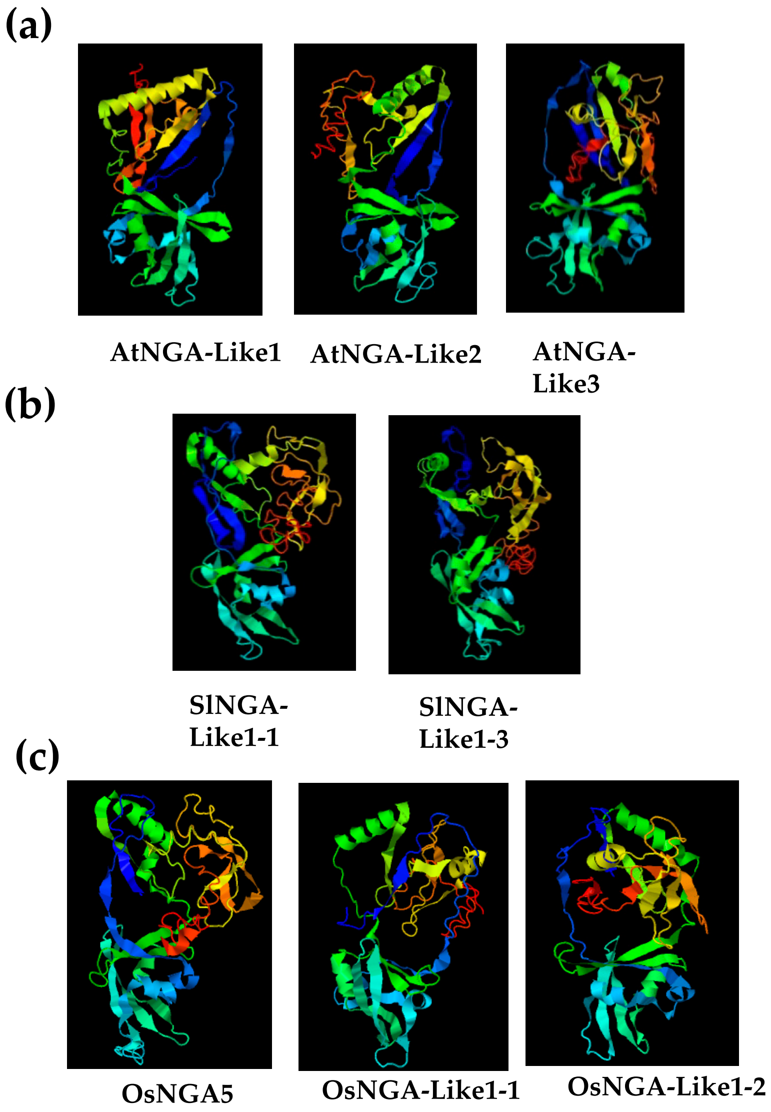


**Figure S6**. 3D-structure of NGATHA-Like proteins in (a) *A. thaliana,* (b) *S. lycopersicum and* (c ) *O. sativa* L. Japonica. The three dimensional structures are acquired using I-TASSER (https://zhanggroup.org/I-TASSER/).

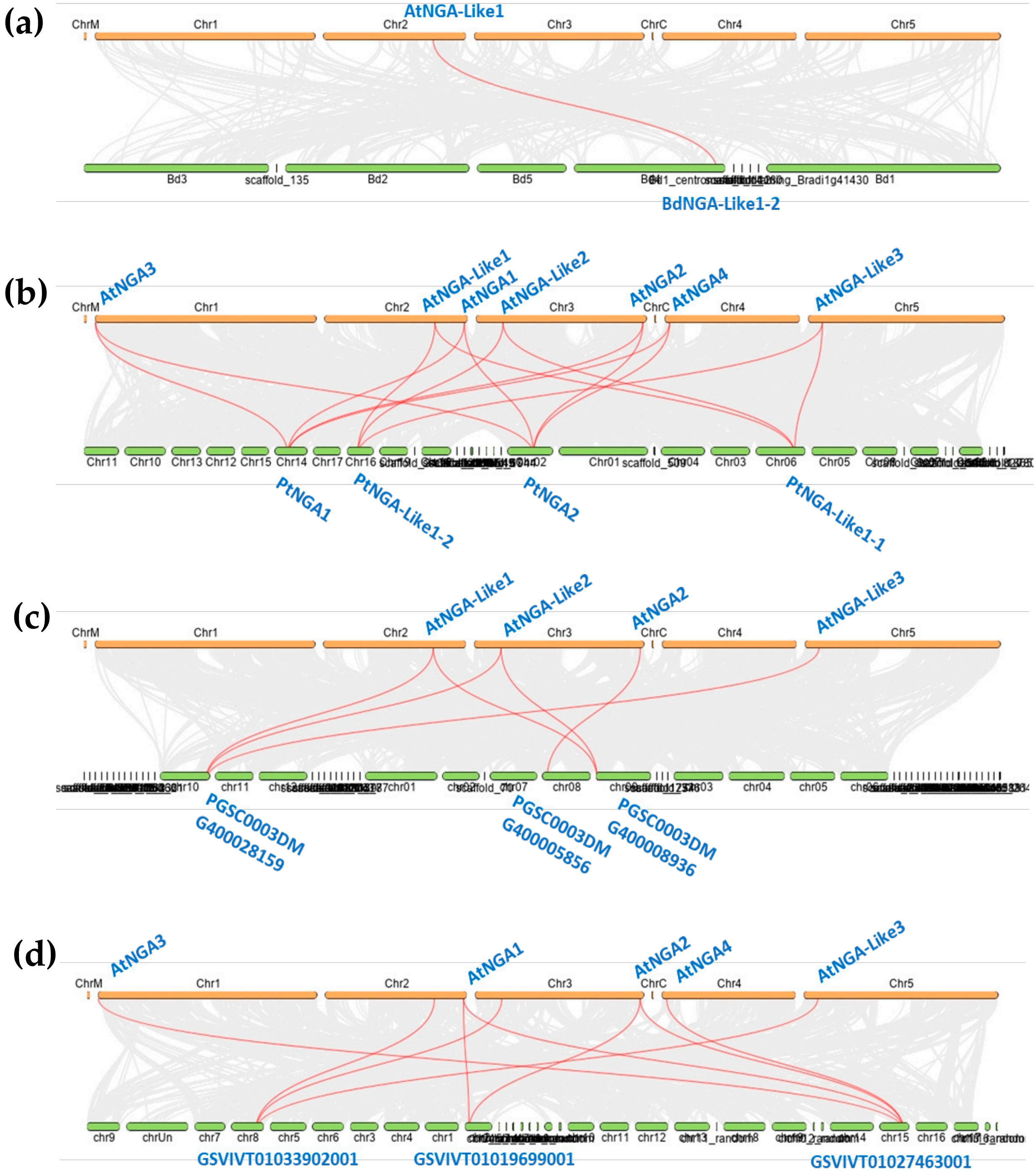


**Figure S7**. Gene duplication or synteny analysis of NGATHA genes in (a) *Brachypodium distachyon*; (b) *Populus trichocarpa;* (c) *S. tuberosum and* (d) *Vitis vinifera* in comparison with *A. thaliana*. The grey lines in the background represent collinear blocks between the respective genomes. The red lines indicate the syntenic gene pairs of *S. lycopersicum, B. rapa and O. sativa* L. Japonica with *A. thaliana*.
