# Supplementary material for: Genome Wide Identification and Annotation of NGATHA Transcription Factor Family in Crop Plants
Source: Int J Mol Sci. 2022 Jun 25;23(13):7063. doi: 10.3390/ijms23137063 (PMC9266525; doi:10.3390/ijms23137063)
Supplement: Supplementary file 1 [file ijms-23-07063-s001.zip › Supplementary tables _IJMS.pdf]

Table S1. List of 207 identified NGATHA genes and related IDs used for phylogeny analysis.

S.No.	Organism	Gene ID	Gene name
**1.**	*Arabidopsis thaliana*	AT2G46870	AtNGA1
**2.**	*Arabidopsis thaliana*	AT3G61970	AtNGA2
**3.**	*Arabidopsis thaliana*	AT1G01030	AtNGA3
**4.**	*Arabidopsis thaliana*	AT4G01500	AtNGA4
**5.**	*Arabidopsis thaliana*	AT2G36080	AtNGA-Like1
**6.**	*Arabidopsis thaliana*	AT3G11580	AtNGA-Like2
**7.**	*Arabidopsis thaliana*	AT5G06250	AtNGA-Like3
**8.**	*Brachypodium distachyon*	BRADI_1g77150v3	BdNGA1
**9.**	*Brachypodium distachyon*	BRADI_3g51840v3	BdNGA2
**10.**	*Brachypodium distachyon*	BRADI_5g19260v3	BdNGA3
**11.**	*Brachypodium distachyon*	BRADI_3g16500v3	BdNGA4
**12.**	*Brachypodium distachyon*	BRADI_3g32140v3	BdNGA5
**13.**	*Brachypodium distachyon*	BRADI_4g25170v3	BdNGA-Like1-1
**14.**	*Brachypodium distachyon*	BRADI_4g42167v3	BdNGA-Like1-2
**15.**	*Brassica rapa*	Bra000434	BrNGA1-1
**16.**	*Brassica rapa*	Bra040478	BrNGA1-2
**17.**	*Brassica rapa*	Bra004501	BrNGA1-3
**18.**	*Brassica rapa*	Bra003482	BrNGA2-1
**19.**	*Brassica rapa*	Bra014415	BrNGA2-2
**20.**	*Brassica rapa*	Bra007646	BrNGA2-3
**21.**	*Brassica rapa*	Bra033294	BrNGA3-1
**22.**	*Brassica rapa*	Bra030497	BrNGA3-2
**23.**	*Brassica rapa*	Bra008552	BrNGA4-1
**24.**	*Brassica rapa*	Bra037417	BrNGA4-2
**25.**	*Brassica rapa*	Bra005301	BrNGA-Like1-1
**26.**	*Brassica rapa*	Bra041023	BrNGA-Like2-1
**27.**	*Brassica rapa*	Bra034828	BrNGA-Like2-2
**28.**	*Brassica rapa*	Bra001411	BrNGA-Like2-3
**29.**	*Brassica rapa*	Bra005886	BrNGA-Like3-1
**30.**	*Brassica rapa*	Bra009171	BrNGA-Like3-2
**31.**	*Beta vulgaris*	BVRB_5g104330	BvNGA1
**32.**	*Beta vulgaris*	BVRB_7g158660	BvNGA-Like1

**33.**	*Capsicum annum*	CA01g34800	CaNGA1
**34.**	*Capsicum annum*	CA01g00060	CaNGA3
**35.**	*Capsicum annum*	CA10g17390	CaNGA-Like1-1
**36.**	*Capsicum annum*	CA09g00050	CaNGA-Like1-2
**37.**	*Camelina sativa*	Csa04g066000	CsNGA1-1
**38.**	*Camelina sativa*	Csa05g002280	CsNGA1-2
**39.**	*Camelina sativa*	Csa06g053390	CsNGA1-3
**40.**	*Camelina sativa*	Csa05g094180	CsNGA2-1
**41.**	*Camelina sativa*	Csa07g002630	CsNGA2-2
**42.**	*Camelina sativa*	Csa16g002690	CsNGA2-3
**43.**	*Camelina sativa*	Csa03g002200	CsNGA3-1
**44.**	*Camelina sativa*	Csa11g027850	CsNGA3-2
**45.**	*Camelina sativa*	Csa12g042130	CsNGA3-3
**46.**	*Camelina sativa*	Csa14g002160	CsNGA3-4
**47.**	*Camelina sativa*	Csa17g001510	CsNGA3-5
**48.**	*Camelina sativa*	Csa02g004620	CsNGA4-1
**49.**	*Camelina sativa*	Csa08g053000	CsNGA4-2
**50.**	*Camelina sativa*	Csa13g055780	CsNGA4-3
**51.**	*Camelina sativa*	Csa01g012670	CsNGA-Like2-1
**52.**	*Camelina sativa*	Csa15g015410	CsNGA-Like2-2
**53.**	*Camelina sativa*	Csa19g015730	CsNGA-Like2-3
**54.**	*Camelina sativa*	Csa08g058280	CsNGA-Like3-1
**55.**	*Camelina sativa*	Csa13g008230	CsNGA-Like3-2
**56.**	*Camelina sativa*	Csa20g008460	CsNGA-Like3-3
**57.**	*Daucus carota*	DCAR 006572	DcNGA1
**58.**	*Daucus carota*	DCAR_020732	DcNGA-Like1-1
**59.**	*Daucus carota*	DCAR 013418	DcNGA-Like1-2
**60.**	*Eucalyptus grandis*	EUGRSUZ L00348	EgNGA1
**61.**	*Eucalyptus grandis*	EUGRSUZ E01093	EgNGA2
**62.**	*Eucalyptus grandis*	EUGRSUZ A02625	EgNGA-Like
**63.**	*Glycine max*	GLYMA_19G261300	GmNGA1-1
**64.**	*Glycine max*	GLYMA_03G262200	GmNGA1-2
**65.**	*Glycine max*	GLYMA_16G017100	GmNGA1-3
**66.**	*Glycine max*	GLYMA_07G048200	GmNGA1-4

**67.**	*Glycine max*	GLYMA_03G199000	GmNGA-Like1-1
**68.**	*Glycine max*	GLYMA_19G196600	GmNGA-Like1-2
**69.**	*Glycine max*	GLYMA_02G200500	GmNGA-Like1-3
**70.**	*Glycine max*	GLYMA_10G076100	GmNGA-Like1-4
**71.**	*Gossypium raimondii*	B456_008G132400	GrNGA1-1
**72.**	*Gossypium raimondii*	B456_001G150200	GrNGA1-2
**73.**	*Gossypium raimondii*	B456_007G114200	GrNGA1-3
**74.**	*Gossypium raimondii*	B456 004G128200	GrNGA1-4
**75.**	*Gossypium raimondii*	B456 003G073600	GrNGA1-5
**76.**	*Gossypium raimondii*	B456 008G261100	GrNGA1-6
**77.**	*Gossypium raimondii*	B456_002G237900	GrNGA-Like1-1
**78.**	*Gossypium raimondii*	B456_009G399000	GrNGA-Like1-2
**79.**	*Gossypium raimondii*	B456_006G152400	GrNGA-Like1-3
**80.**	*Helianthus annuus*	HannXRQ Chr03g0083171	HaNGA1-1
**81.**	*Helianthus annuus*	HannXRQ Chr17g0534081	HaNGA1-2
**82.**	*Helianthus annuus*	HannXRQ Chr06g0167091	HaNGA1-3
**83.**	*Helianthus annuus*	HannXRQ Chr09g0238331	HaNGA-Like
**84.**	*Hordeum vulgare*	HORVU4Hr1G084950	HvNGA1-1
**85.**	*Hordeum vulgare*	HORVU7Hr1G046950	HvNGA1-2
**86.**	*Hordeum vulgare*	HORVU6Hr1G066190	HvNGA2
**87.**	*Hordeum vulgare*	HORVU7Hr1G002230	HvNGA4-1
**88.**	*Hordeum vulgare*	HORVU0Hr1G022200	HvNGA4-2
**89.**	*Hordeum vulgare*	HORVU5Hr1G040620	HvNGA-Like
**90.**	*Musa acuminata*	GSMUA_Achr4G26430_001	MaNGA1-1
**91.**	*Musa acuminata*	GSMUA_Achr4G30170_001	MaNGA1-2
**92.**	*Musa acuminata*	GSMUA_Achr5G12850_001	MaNGA3-1
**93.**	*Musa acuminata*	GSMUA_Achr2G00400_001	MaNGA3-2
**94.**	*Musa acuminata*	GSMUA_Achr1G03600_001	MaNGA3-3
**95.**	*Musa acuminata*	GSMUA_Achr5G20340_001	MaNGA3-4
**96.**	*Musa acuminata*	GSMUA_Achr5G10220_001	MaNGA3-5

**97.**	*Musa acuminata*	GSMUA_Achr5G10230_001	MaNGA3-6
**98.**	*Musa acuminata*	GSMUA_Achr4G24970_001	MaNGA3-7
**99.**	*Musa acuminata*	GSMUA_Achr7G04630_001	MaNGA4-1
**100.**	*Musa acuminata*	GSMUA_Achr4G12210_001	MaNGA4-2
**101.**	*Musa acuminata*	GSMUA Achr6G35600_001	MaNGA-Like1-1
**102.**	*Musa acuminata*	GSMUA Achr6G18320_001	MaNGA-Like1-2
**103.**	*Musa acuminata*	GSMUA_Achr3G19210_001	MaNGA-Like1-3
**104.**	*Musa acuminata*	GSMUA_Achr6G13140_001	MaNGA-Like1-4
**105.**	*Musa acuminata*	GSMUA_Achr9G08980_001	MaNGA-Like1-5
**106.**	*Musa acuminata*	GSMUA_Achr9G02600_001	MaNGA-Like1-6
**107.**	*Manihot esculenta*	MANES 05G015100	MeNGA1-1
**108.**	*Manihot esculenta*	MANES 01G238800	MeNGA1-2
**109.**	*Manihot esculenta*	MANES 01G001000	MeNGA3
**110.**	*Manihot esculenta*	MANES_08G029200	MeNGA-Like1-1
**111.**	*Manihot esculenta*	MANES_09G051400	MeNGA-Like1-2
**112.**	*Medicago truncatula*	MTR_7g117455	MtNGA1-1
**113.**	*Medicago truncatula*	MTR_8g023990	MtNGA1-2
**114.**	*Medicago truncatula*	MTR_1g058350	MtNGA-Like1-1
**115.**	*Medicago truncatula*	MTR_7g105370	MtNGA-Like1-2
**116.**	*Nicotiana tabacum*	Nitab4.5_0000799g0050.1	NtNGA1-1
**117.**	*Nicotiana tabacum*	Nitab4.5_0005519g0040.1	NtNGA1-2
**118.**	*Nicotiana tabacum*	Nitab4.5_0015619g0010.1	NtNGA3-1
**119.**	*Nicotiana tabacum*	Nitab4.5_0012411g0010.1	NtNGA3-2
**120.**	*Nicotiana tabacum*	Nitab4.5_0000573g0030.1	NtNGA-Like
**121.**	*Oryza sativa Japonica group*	LOC_Os03g02900.1	OsNGA1
**122.**	*Oryza sativa Japonica group*	LOC_Os02g45850.1	OsNGA2
**123.**	*Oryza sativa Japonica group*	LOC_Os04g49230.1	OsNGA3
**124.**	*Oryza sativa Japonica group*	LOC_Os08g06120.1	OsNGA4
**125.**	*Oryza sativa Japonica group*	LOC_Os10g39190.1	OsNGA5
**126.**	*Oryza sativa Japonica group*	LOC_Os11g05740.1	OsNGA-Like1-1
**127.**	*Oryza sativa Japonica group*	LOC_Os12g06080.1	OsNGA-Like1-2

**128.**	*Populus trichocarpa*	POPTR_014G107200v3	PtNGA1
**129.**	*Populus trichocarpa*	POPTR_002G181600v3	PtNGA2
**130.**	*Populus trichocarpa*	POPTR_001G452200v3	PtNGA3
**131.**	*Populus trichocarpa*	POPTR_011G149700v3	ptNGA4
**132.**	*Populus trichocarpa*	POPTR_006G208100v3	PtNGA-Like1-1
**133.**	*Populus trichocarpa*	POPTR_016G074500v3	PtNGA-Like1-2
**134.**	*Phaseolus vulgaris*	PHAVU_010G120900g	PvNGA1-1
**135.**	*Phaseolus vulgaris*	PHAVU_009G260100g	PvNGA1-2
**136.**	*Phaseolus vulgaris*	PHAVU_007G230700g	PvNGA-Like1-1
**137.**	*Phaseolus vulgaris*	PHAVU_001G193100g	PvNGA-Like1-2
**138.**	*Sorghum bicolor*	SORBI_3001G528200	SbNGA1
**139.**	*Sorghum bicolor*	SORBI_3004G280500	SbNGA2
**140.**	*Sorghum bicolor*	SORBI_3006G190400	SbNGA3
**141.**	*Sorghum bicolor*	SORBI_3007G047500	SbNGA4
**142.**	*Sorghum bicolor*	SORBI_3001G313800	SbNGA5
**143.**	*Sorghum bicolor*	SORBI_3005G041400	SbNGA-Like1-1
**144.**	*Sorghum bicolor*	SORBI_3008G041100	SbNGA-Like1-2
**145.**	*Setaria italica*	SETIT 036864mg	SiNGA1
**146.**	*Setaria italica*	SETIT 020241mg	SiNGA2
**147.**	*Setaria italica*	SETIT 011823mg	SiNGA3
**148.**	*Setaria italica*	SETIT 014362mg	SiNGA4-1
**149.**	*Setaria italica*	SETIT 008509mg	SiNGA4-2
**150.**	*Setaria italica*	SETIT 039972mg	SiNGA5
**151.**	*Setaria italica*	SETIT 027632mg	SiNGA-Like1-1
**152.**	*Setaria italica*	SETIT 010849mg	SiNGA-Like1-2
**153.**	*Solanum lycopersicum*	Solyc08g013700.1.1	SlNGA1
**154.**	*Solanum lycopersicum*	Solyc08g013690.1.1	SlNGA2
**155.**	*Solanum lycopersicum*	Solyc05g004000.1.1	SlNGA3
**156.**	*Solanum lycopersicum*	Solyc10g083210.2.1	SlNGA-Like1-1

**157.**	*Solanum lycopersicum*	Solyc09g010230.2.1	SlNGA-Like1-3
**158.**	*Solanum tuberosum*	PGSC0003DMP400010383	StNGA1
**159.**	*Solanum tuberosum*	PGSC0003DMP400010384	StNGA2
**160.**	*Solanum tuberosum*	PGSC0003DMP400021619	StNGA3
**161.**	*Solanum tuberosum*	PGSC0003DMP400048918	StNGA-Like1-1
**162.**	*Solanum tuberosum*	PGSC0003DMP400048917	StNGA-Like1-2
**163.**	*Solanum tuberosum*	PGSC0003DMP400015721	StNGA-Like1-3
**164.**	*Solanum tuberosum*	PGSC0003DMP400015722	StNGA-Like1-4
**165.**	*Triticum aestivum*	TraesCS4B02G348500	TaNGA1-1
**166.**	*Triticum aestivum*	TraesCS5A02G516600	TaNGA1-2
**167.**	*Triticum aestivum*	TraesCS6B02G286900	TaNGA2-1
**168.**	*Triticum aestivum*	TraesCS6A02G259600	TaNGA2-2
**169.**	*Triticum aestivum*	TraesCS6D02G240600	TaNGA2-3
**170.**	*Triticum aestivum*	TraesCS2A02G394700	TaNGA3-1
**171.**	*Triticum aestivum*	TraesCS2D02G392400	TaNGA3-2
**172.**	*Triticum aestivum*	TraesCS2B02G412700	TaNGA3-3
**173.**	*Triticum aestivum*	TraesCS7D02G448900	TaNGA4-1
**174.**	*Triticum aestivum*	TraesCS7A02G461000	TaNGA4-2
**175.**	*Triticum aestivum*	TraesCS7B02G362500	TaNGA4-3
**176.**	*Triticum aestivum*	TraesCS7A02G020800	TaNGA4-4
**177.**	*Triticum aestivum*	TraesCS7A02G020700	TaNGA4-5
**178.**	*Triticum aestivum*	TraesCS7D02G017000	TaNGA4-6
**179.**	*Triticum aestivum*	TraesCS4A02G472700	TaNGA4-7
**180.**	*Triticum aestivum*	TraesCS1A02G200500	TaNGA5-1
**181.**	*Triticum aestivum*	TraesCS1B02G215000	TaNGA5-2
**182.**	*Triticum aestivum*	TraesCS1D02G203900	TaNGA5-3
**183.**	*Triticum aestivum*	TraesCS4D02G114900	TaNGA-Like1-1
**184.**	*Triticum aestivum*	TraesCS4B02G117100	TaNGA-Like1-2
**185.**	*Triticum aestivum*	TraesCS4A02G198300	TaNGA-Like1-3

**186.**	*Triticum aestivum*	TraesCS5B02G126200	TaNGA-Like1-4
**187.**	*Triticum aestivum*	TraesCS5A02G127100	TaNGA-Like1-5
**188.**	*Triticum aestivum*	TraesCS2A02G460700	TaNGA-Like1-6
**189.**	*Triticum aestivum*	TraesCS2D02G460900	TaNGA-Like1-7
**190.**	*Triticum aestivum*	TraesCS2B02G482400	TaNGA-Like1-8
**191.**	*Vitis vinifera*	GSVIVT01027463001	VvNGA1
**192.**	*Vitis vinifera*	GSVIVT01019699001	VvNGA3
**193.**	*Vitis vinifera*	GSVIVT01033902001	VvNGA-Like
**194.**	*Zea mays*	Zm00001d027409	ZmNGA1-1
**195.**	*Zea mays*	Zm00001d010077	ZmNGA1-2
**196.**	*Zea mays*	Zm00001d048815	ZmNGA1-3
**197.**	*Zea mays*	Zm00001d017618	ZmNGA2-1
**198.**	*Zea mays*	Zm00001d051471	ZmNGA2-2
**199.**	*Zea mays*	Zm00001d026005	ZmNGA3-1
**200.**	*Zea mays*	Zm00001d002562	ZmNGA3-2
**201.**	*Zea mays*	Zm00001d024545	ZmNGA4-1
**202.**	*Zea mays*	Zm00001d024103	ZmNGA4-2
**203.**	*Zea mays*	Zm00001d035903	ZmNGA4-3
**204.**	*Zea mays*	Zm00001d047359	ZmNGA5-1
**205.**	*Zea mays*	Zm00001d029749	ZmNGA5-2
**206.**	*Zea mays*	Zm00001d052591	ZmNGA-Like1-1
**207.**	*Zea mays*	Zm00001d023446	ZmNGA-Like1-2

Table S2. The similarity matrix of NGA proteins of *A. thaliana*, *S. lycopersicum* and *O.sativa* L. Japonica

**AtNGA1**	100%																		
**AtNGA2**	68.22%	100%																	
**AtNGA3**	54.51%	52.17%	100%																
**AtNGA4**	47.41%	46.15%	52.25%	100%															
**SlNGA1**	48.97%	45.57%	51.36%	44.21%	100%														
**SlNGA2**	49.03%	47.49%	50.3%	43.2%	80.95%	100%													
**SlNGA3**	57.41%	53.17%	44.69%	43.24%	60.88%	58.95%	100%												
**OsNGA1**	45.8%	47.15%	49.19%	45.33%	47.95%	46.62%	47.9%	100%											
**OsNGA2**	46.12%	45.15%	42.17%	40.54%	48.63%	48.14%	43.12%	57.87%	100%										
**OsNGA3**	37.41%	38.12%	39.24%	34.17%	42.17%	38.92%	42.08%	45.98%	62.97%	100%									
**OsNGA4**	41.81%	38.67%	42.5%	38.67%	39.37%	40.76%	38.32%	46.68%	47.73%	39.02%	100%								
**OsNGA5**	42.9%	44.81%	44.55%	42.3%	44.55%	45.19%	44.87%	60.77%	53.2%	43.26%	42.5%	100%							
**AtNGA-Like1**	47.95%	43.03%	47.54%	42.21%	43.85%	47.54%	47.13%	44.67%	47.54%	40.98%	44.67%	43.85%	100%						
**AtNGA-Like2**	45.31%	41.57%	44.94%	39.7%	43.07%	42.69%	40.82%	45.31%	42.32%	38.95%	38.57%	44.19%	57.37%	100%					
**AtNGA-Like3**	40.07%	37.23%	42.9%	39.36%	39.36%	40.78%	41.13%	41.84%	42.55%	37.94%	36.17%	41.84%	59.83%	62.54%	100%				
**SlNGA-like1-1**	36.12%	36.12%	39.87%	35.12%	38.77%	37.97%	39.24%	35.69%	39.24%	35.12%	36.58%	34.93%	61.06%	52.43%	51.41%	100%			
**SlNGA-like1-3**	42.25%	40.8%	37.97%	36.93%	40.81%	39.19%	36.52%	40.19%	37.1%	36.7%	38.32%	39.42%	61.88%	54.68%	53.9%	67.72%	100%		
**OsNGA-Like1-1**	39.78%	39.42%	40.86%	37.63%	39.06%	43.36%	43.72%	46.95%	43.72%	37.63%	37.63%	43.01%	49.18%	43.82%	42.29%	43.36%	45.16%	100%	
**OsNGA-Like1-2**	40.79%	40.43%	41.51%	37.9%	38.98%	41.15%	42.59%	46.93%	42.59%	38.62%	36.46%	41.87%	50%	46.44%	44.76%	45.12%	47.29%	79.06%	100%
	**AtNGA1**	**AtNGA2**	**AtNGA3**	**AtNGA4**	**SlNGA1**	**SlNGA2**	**SlNGA3**	**OsNGA1**	**OsNGA2**	**OsNGA3**	**OsNGA4**	**OsNGA5**	**AtNGA-Like1**	**AtNGA-Like2**	**AtNGA-Like3**	**SlNGA-like1-1**	**SlNGA-like1-3**	**OsNGA-Like1-1**	**OsNGA-Like1-2**

Table S3. List of primers used for qPCR.

Gene name	Forward primer (5′→ 3′)	Reverse primer (5′→ 3′)
AtNGA1	TTGATATGGAATGCGGCGAGAGC	GCAAACTTCCACCGGAAGATGATG
AtNGA2	TCAGCAATTCGCCGGAGCTATG	TTGTCGGCATCTGAGGATGAGG
AtNGA3	ACGGCAAGATGTGGAGATTCCG	CGGCTCCATCCTTTGGTCATAACG
AtNGA4	AGAAACTCTTCGCCGGAGACAC	TCCGGTTGTGCGTTATCGTCAG
AtNGA-LIKE 1	ATCCTCCTCCGTACTACATGGAC	TCTAGCTCGTCCGGTTCATATCTC
AtNGA-LIKE 2	GCTGTCTCCGTTGTGTCTCAAG	TAGTCAAGCCGCTCCAATACGC
AtNGA-LIKE 3	CGCCGTGAATACGAGTTCTATGGG	GGCTCTAGTGGCGTGGATTTGATG
SlNGA1	AAACTTGATGCTGGTGATGTTG	AGTCAATGAAGAGACGGTGC
SlNGA2	CGGAGAAGTATTTCCCTCTTG	CCATGGCTTCCCATTACTATC
SlNGA3	TGTCTCATTTCAGCGTGGAG	CTGGGTACTGAATATTAGAGCGG
SlNGA-LIKE1-1	CTCCGAAGCAAAACAGCAATC	CAACATCACTCGGCGTTAATG
SlNGA-LIKE1-3	AAATATTGGCGGAGGTGGAG	GACAGTCAGGTTGGTATGGAAG
ACTIN	TGTCCCTATTTACGAGGGTTATGC	AGTTAAATCACGACCAGCAAGAT
UBIQUITIN	GCCGACTACAACATCCAGAAGG	TGCAACACAGCGAGCTTAACC
RNaseH	ACTCTCGAAGCTCCACAAGGAAG	AAGCCACTTCAACTTCTCCTTCAG
